# Functionally similar genes exhibit comparable/similar time-course expression kinetics in the UV-induced photoaged mouse model

**DOI:** 10.1371/journal.pone.0290358

**Published:** 2023-11-09

**Authors:** Seon-Pil Jin, Joong Heon Suh, Chang-Eop Kim, Inn Gyung Oh, Eun Young Seo, Min-Kyoung Kim, Kyeong-No Yoon, Jin Ho Chung

**Affiliations:** 1 Department of Dermatology, Seoul National University Hospital, Seoul, Republic of Korea; 2 Department of Dermatology, College of Medicine, Seoul National University, Seoul, Republic of Korea; 3 Institute of Human-Environment Interface Biology, Medical Research Center, Seoul National University, Seoul, Republic of Korea; 4 Department of Biomedical Sciences, Graduate School, Seoul National University Graduate School, Seoul, Republic of Korea; 5 Department of Physiology, Department of Physiology, Gachon University College of Korean Medicine, Seongnam, Republic of Korea; POSTECH - Pohang University of Science and Technology, REPUBLIC OF KOREA

## Abstract

Skin photoaging induced by ultraviolet (UV) irradiation contributes to the formation of thick and coarse wrinkles. Humans are exposed to UV light throughout their lives. Therefore, it is crucial to determine the time-sequential effects of UV on the skin. In this study, we irradiated the mouse back skin with UV light for eight weeks and observed the changes in gene expressions via microarray analysis every week. There were more downregulated genes (514) than upregulated genes (123). The downregulated genes had more functional diversity than the upregulated genes. Additionally, the number of downregulated genes did not increase in a time-dependent manner. Instead, time-dependent kinetic patterns were observed. Interestingly, each kinetic cluster harbored functionally enriched gene sets. Since collagen changes in the dermis are considered to be a major cause of photoaging, we hypothesized that other gene sets contributing to photoaging would exhibit kinetics similar to those of the collagen-regulatory genes identified in this study. Accordingly, co-expression network analysis was conducted using 11 well-known collagen-regulatory seed genes to predict genes with similar kinetics. We ranked all downregulated genes from 1 to 504 based on their expression levels, and the top 50 genes were suggested to be involved in the photoaging process. Additionally, to validate and support our identified top 50 gene lists, we demonstrated that the genes (*FN1*, *CCDC80*, *PRELP*, and *TGFBR3*) we discovered are downregulated by UV irradiation in cultured human fibroblasts, leading to decreased collagen levels, which is indicative of photoaging processes. Overall, this study demonstrated the time-sequential genetic changes in chronically UV-irradiated skin and proposed 50 genes that are involved in the mechanisms of photoaging.

## Introduction

Changes in the skin are easily recognizable signs of aging, but the complex underlying biochemical interactions involved in this process are not yet fully understood. Skin aging leads to wrinkle formation, elasticity reduction, dryness, epidermal thinning, dysregulated pigmentation, and cancer development [1]. Skin aging is classified into two types: intrinsic (chronological) and extrinsic (environmental) aging [2]. Intrinsic aging, which manifests as fine and shallow wrinkles, is influenced by a range of factors, including genetic predisposition, variations in hormone expression, and the length of telomeres [3]. Extrinsic aging is caused by lifestyle-modifiable factors, such as ultraviolet (UV) radiation, visible/infrared light, and air pollutant exposure [4–6]. UV radiation is considered the most influential extrinsic factor that causes photodamage to the skin, leading to accelerated aging, also known as photoaging [7]. Chronic UV exposure not only leads to the formation of coarse and deep wrinkles but also increases the risk of skin cancers, including melanoma, basal cell carcinoma, and squamous cell carcinoma [8].

Wrinkling is a major sign of photoaging, so the literature regarding photoaging mostly describes the dermal extracellular matrix (ECM) changes, mainly collagen, due to its abundance in the dermis at homeostasis and its substantial loss in photoaged skin [3, 7, 9, 10]. However, intracellular changes are essential in regulating the ECM changes of photoaged skin [10]. UV irradiation activates the transcription factor AP-1 in keratinocytes and fibroblasts, promoting the expression of MMPs and degrading collagen in the extracellular matrix, while also reducing TGF-β signaling, which results in photoaging [11]. UV-mediated DNA damage enhances MMP expression to promote photoaging in an aryl hydrocarbon receptor- and specificity protein 1-dependent manner [12]. Despite the numerous pathways previously reported, a more comprehensive understanding of the photoaging skin dynamics is necessary. UV radiation’s ability to target a broad spectrum of surface receptors results in the activation of several downstream signaling pathways, which can significantly influence the expression of multiple genes [13, 14]. Therefore, conducting a more comprehensive investigation of these intricate processes is essential to unravel the complexities of photoaging.

In recent years, several high-throughput studies utilizing single-cell transcriptomics have identified changes in the senescence of human skin. [15, 16] and mouse skin [13]. However, it is difficult to explain whether the results are due to intrinsic or extrinsic aging. Moreover, the effect of UV radiation itself over time in the photoaging process is not completely understood. Therefore, in this study. we established a UV-induced photoaging mouse model and performed various analytical methods including microarray analysis, cluster analysis and network analysis, to identify the time-sequential gene changes significantly involved in photoaging skin.

## Materials and methods

### UV induced photoaging mouse model

All animal experiments were carried out in strict accordance with the recommendations in the Guide for the Care and Use of Laboratory Animals of the National Institutes of Health. The protocol was approved by the Seoul National University Hospital Institute of Animal Care and Use Committee (Protocol Number: 13–0319). Six-week-old female albino hairless mice (Hos:HR-1) were obtained from the SLC (Hizuoka Institute for Laboratory Animals, Inc., Hamamatsu, Shizuoka Prefecture, Japan). The animals were acclimated for one week before the study and had free access to food and water. Three mice were allocated to each group (six groups including the control group). The mice were anesthetized with isoflurane before UV irradiation. A UV irradiation device that included a TL20W/12RS UV lamp with an emission spectrum between 275 and 380 nm (peak: 310–315 nm) was used as the UV source ^1^. A Kodacel filter (TA401/407; Kodak, Rochester, NY, USA) was mounted 2 cm in front of the UV lamp to remove UVC wavelengths of ≤ 290 nm. Irradiation intensity on the mouse dorsal skin was measured using a UV meter (Model 585100; Waldmann Co., Villingen-Schwenningen, Germany). The irradiation intensity 30 cm from the light source was 1.0 mW cm^-2^. Initially, we determined the minimal erythemal dose (MED) in the dorsal skin of mice. MED is the minimum radiation exposure required to produce erythema with sharp margins after 24 h. Irradiation was performed thrice weekly (Monday, Wednesday, and Friday). The UV dose was increased weekly by 1 MED (1 MED = 100 mJ/cm^2^) up to 4 MED and then maintained at this level. UV irradiation was stopped after eight weeks of irradiation. After the experiment, the mice were sacrificed by cervical spine dislocation under anesthesia with isoflurane inhalation.

### Measurement of skin thickness

The dorsal skin of hairless mice was lifted up by gentle pinching, and skin-fold thickness was measured using a caliper (Peacock; Ozaki MFG Co. Ltd., Tokyo, Japan). Skin thickness was measured at weeks 0, 1, 2, 4, 6, and 8.

### Evaluation of wrinkle formation

Skin wrinkle replicas were prepared by applying silicon rubber (Silflo Dental Impression Material; Flexico Developments, Stevenage, Hertford-shire, UK) to the dorsal skin of the mice. Skin impressions were photographed using a coupling charge system video camera and analyzed using the Skin-Visiometer SV 600 software (Courage + Khazaka electronic GmbH, Köln, Germany).

### Statistical analysis

Bars in the Figures indicate the mean ± standard deviation of n = 6 animals per group. Data were statistically analyzed via one-way analysis of variance followed by Duncan test and considered significantly different when *P* < 0.05.

### Masson’s trichrome staining

Skin samples were fixed with 4% PFA and paraffin-embedded, and sections were prepared with 4um thickness. The sections were deparaffinized, hydrated in ethanol, and subjected to heat induced antigen retrieval in 0.01 mol/L citrate buffer (pH 6.0). Masson’s trichrome stain was performed using standard protocols.

Collagen density quantification was performed using Image J software with the "Colour Deconvolution2" plugin. Mean collagen density was determined for each section based on measurements taken from three randomly selected square areas within the papillary dermis.

### Microarray analysis

Total RNA was isolated from mouse skin using RNAiso Plus reagent (Takara Bio Inc.) following the manufacturer’s protocol. cDNA was synthesized using the RevertAid First Strand cDNA Synthesis Kit (Thermo Fisher Scientific). Synthesized cRNA from each tissue was labeled according to the manufacturer’s recommendations and hybridized onto Affymetrix Mouse Genome 430 2.0 arrays (Affymetrix, Santa Clara, CA), which covered more than 45,000 transcripts. CEL files produced by the GeneChip Operating System (Affymetrix) were imported into the Affymetrix Expression Console Software (version 1.4) using a Microarray Suite 5 normalization preprocessor. Probe sets with normalized values in all samples exhibiting less than 20% intensity were filtered out, and those with “absent” detection calls in all samples were also excluded. Photoaged skin samples were then compared with non-irradiated samples to identify the up- and downregulated genes with two-fold or greater changes in expression levels (Benjamini–Hochberg false-discovery rate [FDR] ≤ 5%). The dataset is accessible on the Gene Expression Omnibus database (series accession number: GSE58915).

Genes that showed at least 2-fold differential expression at one time point were included for further analysis. Up- and downregulated genes were analyzed separately. K-means clustering analysis was conducted using the MultiExperiment Viewer (MeV ver. 4.9) software.

The official gene symbols were used as input data for DAVID Bioinformatics Resources 6.7. Functional annotation was performed for the Gene Ontology (GO) biological process, molecular function, and cellular component categories. Systematic and integrative analyses of large gene lists were conducted using DAVID bioinformatics resources [17].

### Quantitative reverse transcription-quantitative polymerase chain reaction (RT-qPCR) analysis

Expression changes were validated using quantitative PCR on cDNA from UV-irradiated and non-irradiated tissues using an ABI prism 7500 sequence detector (Applied Biosystems, Foster City, CA) with SYBR Green mix (TaKaRa Bio, Shiga, Japan). RNA was extracted using the RNAiso Plus reagent (TaKaRa Bio, Shiga, Japan). RNA (2 μg) was used for reverse transcription using a Revert-Aid cDNA synthesis kit (MBI Fermentas, Glen Burnie, MD), and one-tenth of the reaction was used as a template in PCR. All primer sets were run under the following cycling conditions: 95°C for 3 min, followed by 40 cycles of 95°C for 10 s, and annealing at 60°C for 45 s, with data acquisition during each cycle. Melting curve analysis following PCR cycling was used to determine the purity and quality of PCR products. Relative mRNA quantification was performed using the comparative ΔΔCt method. The specific primer sequences used in this study are listed in S1 Table.

### Co-expression network analysis

#### Network construction

Nodes and edges of the co-expression networks were defined as differentially expressed genes (DEGs) and correlation coefficients among genes, respectively. Pearson correlation coefficients were computed between the 637 upregulated and downregulated genes using time-dependent changes to construct networks. The network was represented by a 637 × 637 matrix, in which each element indicated the value of the edge between the corresponding nodes (weighted matrix). Then, the matrix was binarized to a number of adjacency matrices with only one or zero as elements (1 = connected, 0 = unconnected) by applying thresholds in increments of 0.001 from 0.001 to 0.999. The networks were visualized using Cytoscape (www.cytoscape.org).

#### Neighborhood analysis-based candidate gene prediction

We predicted new candidate genes for UV-induced photoaging by identifying the neighbors of collagen regulation genes in binary co-expression networks. Neighboring genes were defined as those directly connected to seed genes in binary co-expression networks. Eleven genes related to collagen biosynthesis or processing that were downregulated were set as the seed genes. A nested leave-one-out-cross-validation (LOOCV) framework was employed to confirm the reliability of the prediction. One seed gene was excluded from the seed list for testing purposes. Using the remaining 10 seed genes, neighborhood scores were evaluated for all genes in the network by adding 1 s for each connection to one seed gene, resulting in every gene having scores ranging from 0 (no connection to seed genes) to 10 (connected to 10 seed genes). Genes were ranked according to the neighborhood score, and the rank of the testing gene was identified. This process was repeated for the 11 seed genes, resulting in their rank evaluation (outer loop).

To determine the optimal network thresholds for each prediction (hyperparameter tuning) while preventing the selection of the thresholds yielding the best prediction performance in LOOCV, an additional inner-loop LOOCV was performed in each evaluation. In each inner-loop validation, binary networks with specific thresholds were chosen (0.001–0.999) in turn. One seed gene was excluded for hyperparameter tuning, and the remaining nine genes were used to evaluate the neighboring scores. The ranks of the test genes were identified. After 999 iterations of inner-loop validation (0.001–0.999), the optimal network threshold for each outer-loop validation was determined. The outlier seed gene (defined as the gene with the highest rank in the inner loop validation above 200 across all thresholds) was excluded from the seed gene list for each outer loop validation. Final candidate gene prediction was performed using all 11 collagen genes as seeds and a network with a median optimal threshold of LOOCV.

#### Cell culture and UV irradiation

Primary human dermal fibroblasts (HDFs) were isolated and cultured using our previous experiment’s protocol [18]. Four distinct strains of HDFs at passages 4–6 were examined. The research protocols involving human subjects were approved by the Seoul National University Institutional Review Board (IRB number: 1101-116-353), and informed consent was obtained from all volunteers, in accordance with the principles outlined in the Declaration of Helsinki.

For cell UV irradiation, after culture in a 60 mm dish, cells were washed with phosphate-buffered saline (PBS) and irradiated with UV light in PBS at 37°C with the culture dish lid opened. Then, the PBS was replaced with growth media. UV irradiation was performed once at a dose of 100 mJ/cm^2^, and the cells were evaluated at 24-, 48-, and 72-hour time points. Philips TL20W/12RS sun lamps with an emission spectrum between 275 and 380 nm (peak, 310–315 nm) were used for UV irradiation, while UVC wavelengths below 290 nm were blocked by the filter. UV intensity was measured using a UV meter (model No. 585100, Waldmann Co., Germany).

#### Transfection of siRNA

For gene silencing of FN1, CCDC80, PRELP, TGFBR3, and LUM, specific siRNAs were used. Negative control siRNA (AccuTarget™ Negative control siRNA) and specific siRNAs targeting *FN1*, *CCDC80*, *PRELP*, *TGFBR3*, and *LUM* were obtained from Bioneer (Republic of Korea) (siRNA No: 2335–1, 4060–1, 5549–1, 7049–1, 151887–1). When human fibroblasts reached 60% confluence, 40nM siRNAs were transfected using G-fectin (Genolution, Republic of Korea) and incubated for 48 hours.

#### Immunoblotting

Collected fibroblasts were lysed in RIPA buffer (BD Biosciences, USA) including protease inhibitors (Roche, Germany) and phosphatase inhibitor (Sigma, USA). The harvested proteins were quantified using bicinchoninic acid assay. A total of 20μg protein extracts were resolved on SDS-polyacrylamide gels and then transferred onto nitrocellulose and polyvinylidene fluoride membranes. After blocking for 1 hour in 5% skim milk, the membrane was incubated overnight with primary antibodies (1:1000) at 4°C. The membranes were then washed and incubated with horseradish peroxidase-linked secondary antibody (1:10,000) for 1 h at room temperature. Immunoreactive proteins were detected using the ECL substrate from Biomax (Korea). The antibodies used include type I procollagen (SP1. D8; N-terminal extension peptide, Developmental Studies Hybridoma Bank, USA) and β-actin (MA5-15739, Thermofisher, USA).

## Results

### Establishment of a mouse model to evaluate UV-induced photoaging of the skin

**Fig 1:**
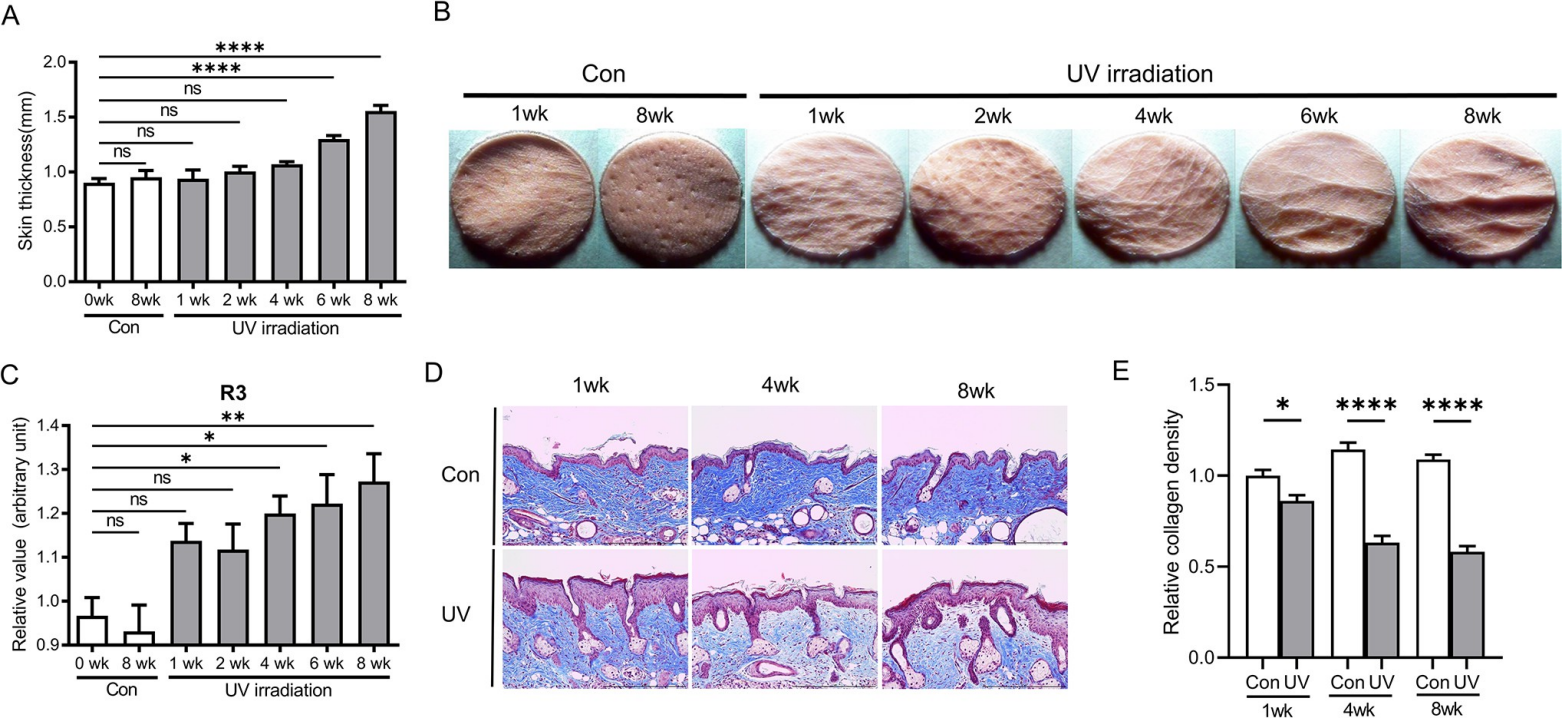
Photoaging mice model was made. (A) Skin fold thickness changes over time. (B) Wrinkle formation was assessed after UV exposure at the independent time points by preparing skin replicas (C) and measured quantitatively by computer scanning of replicas. R3 means ‘Average Roughness’. Data represent mean + s.e.m (n = 6). (D) Masson-trichome staining of 1, 4, 8- week UV exposed mice skin tissues. (E) Quantification of collagen density in skin sections (n = 6). *P*-values were obtained by one-way ANOVA and Duncan multi-comparison test. * *P* < 0.05, ** *P* < 0.01, **** *P* < 0.0001 Con, control; wk, week.

To identify genes that contribute to UV-induced wrinkle formation, we used a murine photoaging model in which chronic low-dose UV irradiation induced wrinkle formation in an animal model [19]. Mice were progressively exposed to UV thrice weekly, starting with 1 MED (100 mJ/cm^2^) per exposure and increasing up to 4 MED (400 mJ/cm^2^), and remaining constant from week 6 to week 8. We examined the effects of photoaging on UV-induced skin thickening and wrinkle formation in our mouse model. To verify and exclude intrinsic aging effect itself, we used both non-UV-exposed 1- and 8 week-mouse skin as controls. Skin fold thickness measured by the caliper was increased in a time-dependent manner compared to that in the non-UV irradiated 1 week control, and statistical significances were observed in week 6 and 8 groups (Fig 1A). Wrinkle scores were determined using a replica and Visiometer. The wrinkle grade R3 values increased significantly after 1 week, and the highest score was observed between weeks 6 and 8 of UV irradiation of the dorsal skin (Fig 1B and 1C). Histologically, collagen expression of papillary dermis was significantly decreased by UV irradiation (Fig 1D and 1E).

### Transcriptional profiling of photoaged mouse skin

Microarray analysis was performed to identify the genes associated with skin photoaging.

**Fig 2:**
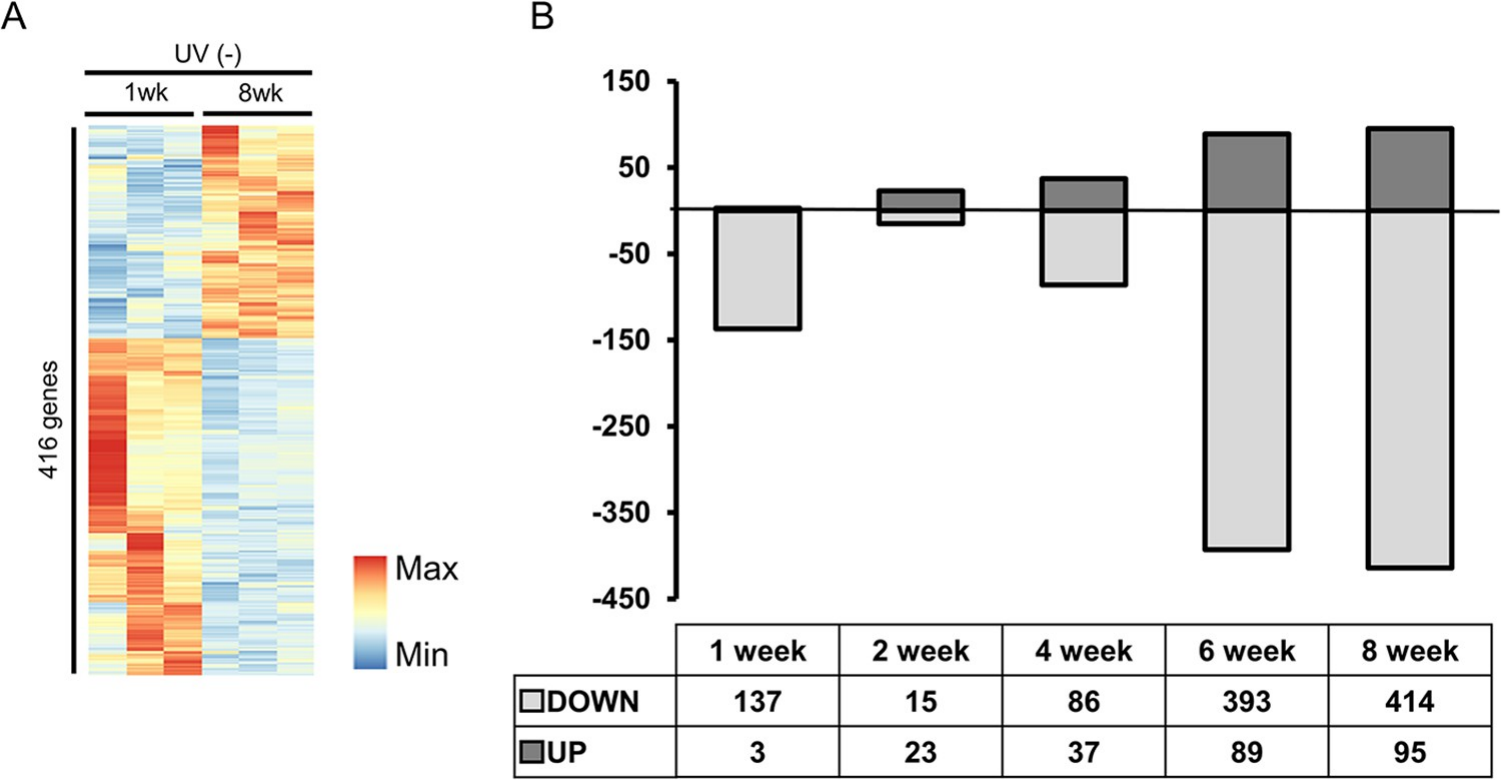
Exploration of up- and down-regulated genes at each UV irradiation time-point. (A) Heatmap comparing the lists of DEGs showing more than 1.5-fold changes in expression levels between UV non-irradiated week 1 and week 8 control mice. (B) The number of DEGs exhibiting over 2-fold changes in expression levels in UV-irradiated mice at weeks 1, 2, 4, 6, and 8, as compared to non-irradiated week 1 control mice.

Fig 1 shows that repeated and accumulated UV irradiation causes wrinkle formation. After 8 weeks of UV exposure, mice reflected chronological changes (aging) compared to the mice at week 1. To mitigate or eliminate the impact of intrinsic chronological changes, we selected genes with minimal changes (<1.5 fold) compared to the 1-week to 8-week control group, while excluding genes with variable changes (>1.5 fold) (Fig 2A). Genes with at least two-fold change at one time point during the 8-week UV exposure were chosen. Based on these analyses, 637 DEGs were identified (Fig 2B). Interestingly, except for week 2, the number of downregulated genes was larger than that of upregulated genes, and the majority of DEGs were found at weeks 6 and 8.

To compare the significant enrichment of functional groups, we subjected the up- and downregulated genes to DAVID analysis. The major functional GO terms of upregulated genes were “lipid biosynthetic process” and “immune response,” while those of down-regulated genes showed more functional diversity, including “glycosaminoglycan binding,” “tissue morphogenesis (epithelium development),” “extracellular matrix,” and “negative regulation of apoptosis” (Table 1).

**Table 1:** Enriched common GO terms among differentially expressed genes by DAVID analysis.

**GO terms**	**Up regulated genes**	**FDR q-value**
lipid biosynthetic process	*Far1*, *Cyp17a1*, *Mboat7*, *Ugcg*, *Lss*, *Elovl7*, *Pdss1*, *Hsd17b7*, *Degs2*	8.9E-04
organic acid biosynthetic process	*Far1*, *Asns*, *Elovl7*, *Gsto1*, *Psat1*, *Degs2*	8.2E-03
carboxylic acid biosynthetic process	Same as ‘organic acid biosynthetic process’ genes	8.2E-03
immune response	*Exo1*, *Ifih1*, *Lst1*, *Serpina3g*, *Oasl2*, *Samhd1*, *Defb1*, *Tlr7*, *Cxcl10*, *Isg15*	2.5E-02
	**Down regulated genes**	
glycosaminoglycan binding	*Smoc2*, *Fgfr1*, *App*, *Ccdc80*, *Tgfbr3*, *Fstl1*, *Adamts1*, *Tpsb2*, *Thbs1*, *Pcolce2*, *Fn1*, *Cyr61*	9.3E-04
polysaccharide binding	‘Glycosaminoglycan binding’ genes + *Enpp2*, *Agl*	1.2E-04
morphogenesis of an epithelium	*Fgfr3*, *Nrp1*, *Lmo4*, *Npnt*, *Smad4*, *Igf1*, *Jag1*, *Pthlh*, *Igf1r*, *Sfrp1*, *Sema3c*, *Tgif1*, *Chuk*	7.8E-03
epithelium development	‘Morphogenesis of an epithelium’ genes + *Gja1*, *Irf6*, *Jun*	1.7E-02
tissue morphogenesis	‘Morphogenesis of an epithelium’ genes + *Twsg1*, *Fkbp1a*, *Tpm1*, *Tgfbr3*, *Col11a1*	3.7E-04
positive regulation of cell communication	*Fgfr1*, *Twsg1*, *Fgfr3*, *Ube3a*, *Npnt*, *Skp2*, *Smd4*, *Jag1*, *Lpar1*, *Ctnna1*, *Igf1r*, *Nras*, *Gan1*, *Thbs1*, *Chuk*	1.3E-03
positive regulation of signal transduction	Same as ‘positive regulation of cell communication’ genes	4.7E-04
extracellular matrix organization	*Smoc2*, *App*, *P4ha1*, *Npnt*, *TgfbrI*, *Ccdc80*, *Col11a1*, *Serpinh1*, *Cyr61*	2.9E-02
proteinaceous extracellular matrix	*Matn2*, *Col4a2*, *Lum*, *Npnt*, *Adamts15*, *Ccdc80*, *Sparc*, *Timp3*, *Prelp*, *Smoc2*, *Gpc6*, *TgfbI*, *Tgfbr3*, *Adamts1*, *Mfap4*, *Col11a1*, *Fn1*	1.4E-02
extracellular matrix	Same as ‘proteinaeous extracellular matrix’ genes	2.1E-02
negative regulation of apoptosis	*Fgfr1*, *Rock1*, *Skp2*, *Bnip3*, *Birc6*, *Igf1*, *Sgms1*, *Ctnna1*, *Ube2b*, *Adora1*, *Nras*, *Bfar*, *Tsc22d3*, *Agtr1a*, *Rasa1*	1.5E-02
negative regulation of cell death	Same as ‘negative regulation of apoptosis’ genes	1.9E-02
negative regulation of programmed cell death	Same as ‘negative regulation of apoptosis’ genes	1.8E-02

To validate the reliability of microarray analysis results, we selected six randomly up- and down-regulated genes from the DEG list (compared to 1 week control) and performed SYBR-based quantitative real-time PCR. *Gasdermin C*, *Isg15*, and serum amyloid A3 (*Saa3*) and KLF transcription factor 4 (*Klf4*), S100 calcium-binding protein A3 (*S100a3*), and tescalcin (*Tesc*) genes were significantly up- and downregulated, respectively, in our photo-aged model (S1 Fig).

### Up- and downregulated DEGs exhibit time-sequential kinetic patterns (clustering analysis)

**Fig 3:**
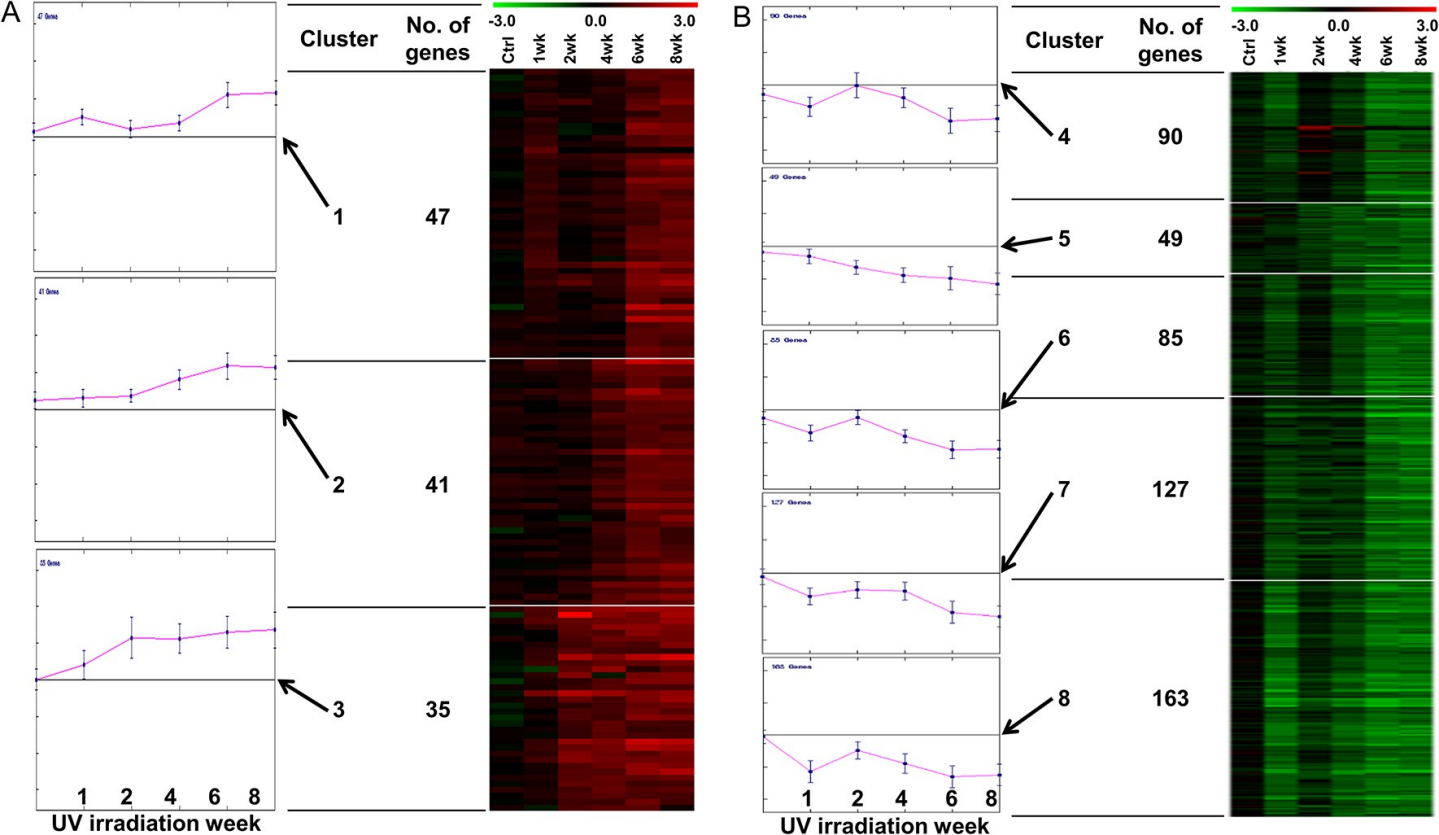
K-means clustering was performed to cluster the genes according to their expression kinetics. (A) Clusters one to three represent kinetics of up-regulated genes, (B) whereas clusters four to eight correspond to five different kinetics of gene repression. The left panel indicates expression levels at each week. Middle points are the number of genes that each cluster contains. The right panel is a heat map to look over the expression pattern of each cluster. Row means individual genes. Columns are time points of analysis. Red indicates upregulation of the gene expressions, and green means downregulated gene expressions.

K-means clustering was performed separately on the up- or downregulated genes. Initial K was used to categorize the genes into up- and downregulated groups. We then obtained a total of eight clusters representing the specific pattern of gene regulation (Fig 3). Clusters 1–3 represent the kinetics of upregulated genes, whereas clusters 4–8 correspond to five different kinetics of downregulated genes.

In cluster 1, 47 transcripts showed acute upregulation in the first week, which returned to near basal levels in the second week, and then upregulated again to peak level from weeks 6 to 8. Functional annotation using DAVID Bioinformatics tools revealed that this group had functional GO term of “immune response” with an FDR q-value of 9.9E-02; exonuclease 1 (*Exo1*), leukocyte-specific transcript 1 (*Lst1*), SAM and HD domain-containing deoxynucleoside triphosphate triphosphohydrolase 1 (*Samhd1*), defensin beta 1 (*Defb1*), and toll-like receptor 7 (*Tlr7*) were included.

Cluster 2 (41 transcripts) comprised genes that responded to repeated UV irradiation after 2 to 4 weeks. This group can be designated as subacute activation in response to repeated UV irradiation.

Cluster 3 (35 transcripts) included genes with induction that started in the first week and then maintained from weeks 2 to 8 during the repeated UV irradiation period.

Clusters 4–8 showed five kinetics of repression following repeated UV irradiation. Cluster 4 (90 transcripts) represented genes that were downregulated in the first week, which were restored or even slightly induced in the second week, and then repressed again after week 4. It belongs to a functional group of the “extracellular matrix” with an FDR value of 2.87E-02. Cluster 5 (49 transcripts) included genes whose expressions decreased continuously over time. In cluster 6 (85 transcripts), genes were downregulated in the first week, recovered to basal levels in the second week, and then repressed again after that. It had GO terms of “glycosaminoglycan binding,” “extracellular matrix organization,” “proteinaceous extracellular matrix,” and FDR q-values of 9.8E-04, 5.6E-03, and 1.5E-02, respectively. Cluster 7 (127 transcripts) showed repression in the first week, and its expression level was maintained until week 4, then continued to decrease again from weeks 6 to 8. Cluster 8 (163 transcripts) had gene expression patterns similar to those of Clusters 4 and 6. However, in contrast to clusters 4 and 6, the expression level of genes in cluster 8 fell much more in the first week, regained but was less than basal levels, and repressed again to a level similar to that in week 1.

Of all the 514 down-regulated genes, a total of 17 genes belonged to the GO annotations of “proteinaceous extracellular matrix” (all are also included in GO of “extracellular matrix”) with FDR q-value of 1.4E-02. Clusters 4 and 6 had very similar expression patterns, and they included 14 of 17 genes of those “proteinaceous extracellular matrix.” Considering that clusters 4 and 6 had only 175 of the 514 downregulated transcripts (34.0%), this was quite intriguing (14/17, 82.4%; fold enrichment = 2.42, chi-square test, *P* < 0.001). In addition to these GO terms, cluster 6 contained some well-known genes for collagen biosynthesis and processing: tropocollagen formation (*Pcolce2*), intrafibril stability (*Leprel2*), collagen (*Col4a2*, *Col11a1*), collagen fibril organization (*Lum*), procollagen transport (*Serpin h1*, *Serpin g1*), and collagen fibrillogenesis (*Sparc*, *Smoc2*) [20, 21].

### Identification and characterization of 50 new candidate genes for UV-induced photoaging via co-expression network analysis

We further investigated the correlated expression patterns among DEGs to identify new candidate genes for UV-induced photoaging. We specifically focused on collagen-regulatory genes, which are known to be involved in the photoaging process. We hypothesized that at least some genes that have similar kinetics to genes involved in collagen regulation may play a role in the regulation of collagen, be involved in the ECM, or contribute to one of the photoaging phenotypes. We constructed co-expression networks of DEGs. We speculated that the neighboring genes of collagen-regulatory genes in the constructed co-expression network could be potential candidate genes for UV-induced photoaging. To confirm the reliability of this approach and determine the optimal threshold for binary network construction, we employed a nested LOOCV framework (see Methods).

**Fig 4:**
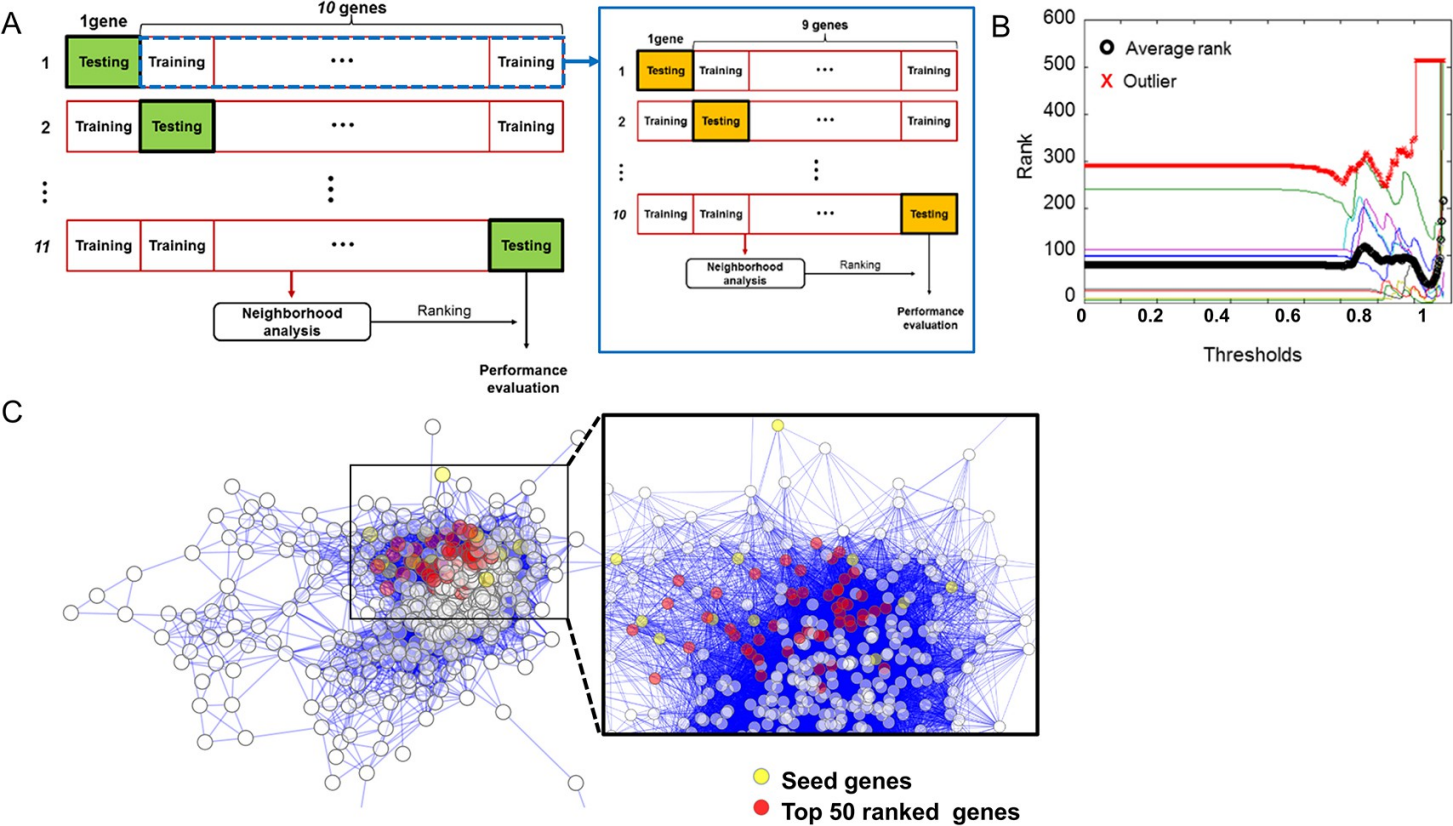
Co-expression network construction to neighborhood analysis-based related gene prediction. (A) Nested leave-one-out-cross-validation was performed. One gene was excluded from each seed gene list in turn for testing and using the remaining 10 genes (training genes) as seeds, neighborhood score was evaluated for all down-regulated DEGs in the co-expression network. Prediction performance was evaluated by ranking the excluded seed gene (green). To determine the best network threshold for prediction, inner loop cross-validation was performed in each fold (right box). Inner loop validation was iterated for networks with varying thresholds. (B) An example of inner loop validation across thresholds (0.001–0.999). Predicted ranks of 10 genes across thresholds were represented as colored traces. A trace of an outlier gene was marked with red cross markers. The averaged rank of 9 genes (except for the outlier) was represented as a black trace with open circles. Note that the best performance was achieved at around 0.95. (C) Constructed co-expression network. Eleven seed genes are yellow circles. Red circles mean genes with top 50 neighborhood score.

Overall, 11 collagen regulation genes were highly ranked in the neighborhood analysis-based candidate gene prediction (median rank 39 among 504 genes) (S2 Table). Significance was tested by permutation testing with 10^7^ repetitions (*P* < 0.0001). Notably, each 11 rank prediction was performed using a co-expression network of downregulated genes and only 10 known collagen regulation genes (as seed genes for neighborhood scoring). However, testing gene information was never used for each prediction, supporting the reliability of our approach for identifying new candidate genes. Finally, we predicted 50 high-ranked candidate genes using the 11 collagen-regulatory genes in the network with the optimal network threshold (0.946). The 50 high-ranked genes, excluding the known 11 genes, are shown in Fig 4 and Table 2.

**Table 2:** Top 50 genes that showed similar kinetics during the 8-week-UV irradiation experiment with 11 collagen regulation genes, and their GO functional annotations by DAVID analysis.

50 candidate genes (in order of ranking)	GO functional annotations	Enriched genes	*P-value
*Qk*, *Il11ra1*, *AU015680*, *Ccdc80*, *Sfrp1*, *Fgfr3*, *Fstl1*, *Axl*, *N/A*, *N/A*, *Prelp*, *Lmo4*, *Fn1*, *Gpr177*, *Pdlim7*, *Cd47*, *Rfx7*, *Rnpc3*, *Chuk*, *Schip1*, *Inhbb*, *Arl6ip5*, *Gpr177*, *Htra1*, *Gatm*, *Gda*, *Vldlr*, *Bpgm*, *Igf1r*, *Enpp2*, *Myadm*, *Sfrs6*, *Sspn*, *N/A*, *Maged1*, *Tgif1*, *Nktr*, *N/A*, *Gnas*, *Ttc7b*, *Lum*, *1700019D03Rik*, *N/A*, *Gnai1*, *Gtf3c2*, *Klf9*, *Serinc1*, *Tgfbr3*, *Zfp280d*, *Pde4dip*	Morphogenesis of an epithelium	*Lmo4*, *Tgif1*, *Chuk*, *Fgfr3*, *Igf1r*, *Sfrp1*	0.001
	Proteinaceous extracellular matrix	*Ccdc80*, *Fn1*, *Lum*, *Prelp*, *Tgfbr3*	0.018
	Polysaccharide binding	*Ccdc80*, *Enpp2*, *Fn1*, *Fstl1*, *Tgfbr3*	0.007
	Enzyme linked receptor protein signaling pathway	*Axl*, *Fgfr3*, *Igf1r*, *Schip1*, *Tgfbr3*	0.007

N/A, not annotated, * *P*-value from Fisher’s exact test

We conducted a GO functional annotation analysis of these 50 genes using DAVID tools. As expected, GO terms associated with “extracellular matrix,” “proteinaceous extracellular matrix,” and “polysaccharide binding” were enriched (fold enrichment = 3.02 and 3.67, respectively). Notably, when we obtained the genes that showed similar expression kinetics with the genes involving “collagen synthesis and processing” during UV irradiation, genes (*Lmo4*, *Tgif1*, *Chuk*, *Fgfr3*, *Igf1r*, *Sfrp1*) that associated with “morphogenesis of epithelium” were enriched (fold enrichment = 4.74, Fisher’s exact test, *P* = 0.001, Table 2).

### Downregulation of *FN1*, *CCDC80*, *PRELP*, and *TGFBR3* gene expressions in human fibroblast induced by UV irradiation leads to collagen reduction

To determine if the genes we identified through network analysis are indeed involved in the photoaging process, we focused on studying the genes associated with the "proteinaceous extracellular matrix" and their role in regulating collagen during photoaging. To investigate their functionality, we conducted experiments using cultured human fibroblasts.

**Fig 5:**
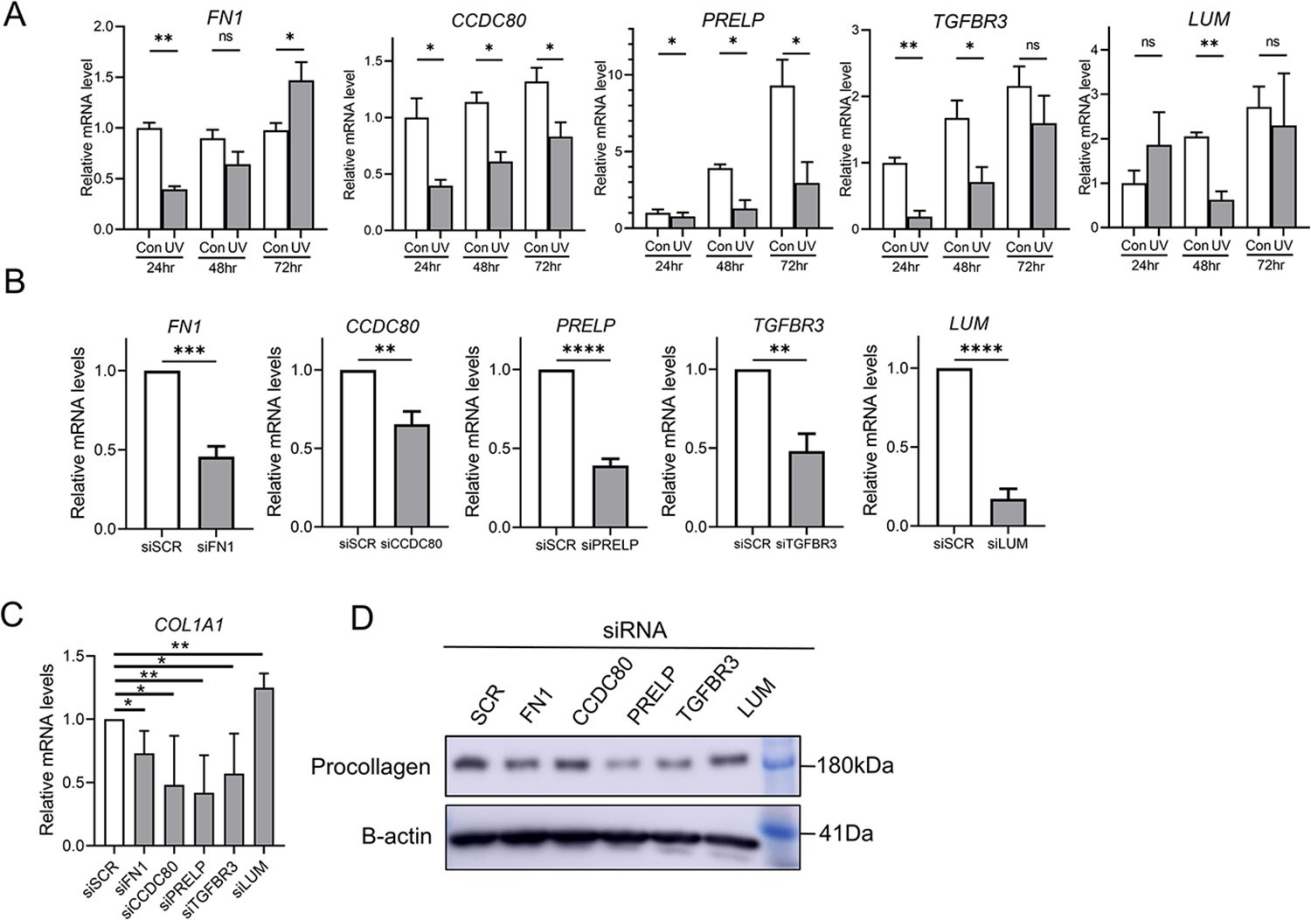
UV irradiation downregulates expressions of *FN1*, *CCDC80*, *PRELP*, *TGFBR3*, and *LUM*, resulting in reduction of collagen synthesis. (A) UV-irradiated and non-irradiated human fibroblasts were harvested at 24, 48, and 72-hour timepoints. mRNA expression levels of *FN1*, *CCDC80*, *PRELP*, *TGFBR3*, and *LUM* were evaluated. (B) Gene-specific siRNA transfections were performed to study the effects of gene knockdown on the target genes. (C) mRNA levels of *COL1A1* were assessed in siRNA-transfected fibroblasts to examine the impact of gene knockdown. (D) Immunoblotting analysis of procollagen in siRNA-transfected cell lysates. Representative image of three independent experiments. Data presented as mean + s.e.m. *P*-values were obtained by Welch’s t test. * *P* < 0.05, ** *P* < 0.01, *** *P* < 0.001, **** *P* < 0.0001. siSCR. Scrambled siRNA; Con. control; hr. hour.

Upon exposing the fibroblasts to UV irradiation, we observed a decrease in the expression levels of *FN1*, *CCDC80*, *PRELP*, *TGFBR3*, and *LUM* (Fig 5A). To understand whether the reduced expression of these genes contributes to collagen downregulation, we performed siRNA transfections targeting these genes, along with a control using scrambled siRNA, in normal human fibroblasts (Fig 5B). Remarkably, our results showed a significant decrease in collagen mRNA expression in fibroblasts transfected with siRNA targeting *FN1*, *CCDC80*, *PRELP*, and *TGFBR3* (Fig 5C). Moreover, we observed a similar decrease in the protein levels of procollagen (Fig 5D).

In summary, these findings strongly indicate that *FN1*, *CCDC80*, *PRELP*, and *TGFBR3*, which are downregulated by UV irradiation, play a crucial role in the downregulation of collagen during the process of photoaging.

## Discussion

Of the many factors determining the skin aging process, UV radiation is the most detrimental, but it is also the most modifiable. Therefore, a comprehensive understanding of the mechanism of skin photoaging caused by UV radiation is critical not only for protecting skin health but also for cosmetic purposes. Chronic UV irradiation induces photoaging phenotypes, such as wrinkling, dehydration, sagging, irregular pigmentation, and tumor development [22, 23] by affecting the epidermis, dermis, and even the subcutaneous fat tissue. We focused on wrinkle formation, a prominent visible change, and a representative phenotype of chronic UV exposure on the skin.

Although many studies have extensively investigated photoaging, [11–13] the molecular mechanisms by which chronic UV irradiation affects skin biology and determines the clinical phenotypes, including wrinkle formation, remain unclear. In addition, there have been no studies on photoaging using a time-dependent photoaging model. In this study, we optimized a UV-induced time-dependent photoaging model and employed microarray analysis to obtain a comprehensive view of the changes in gene expression levels during the photoaging process. UV radiation doses were selected to prevent excessive skin damage, such as erosion or blister formation, which are not observed in chronic human skin photoaging. Also, UV doses were gradually increased to deliver sufficient UV effect to dermis penetrating the thickened epidermis. Our mouse model showed prominent wrinkle formation and increased skin thickness after 6–8 weeks of UV irradiation. Consistent with this result, maximum modulation of gene expression was observed at 6–8 weeks in mouse models via microarray analysis.

Interestingly, the number of downregulated DEGs did not increase in a time-dependent manner; instead, it showed some kinetic pattern, while we increased the UV dose from 1 to 4 MED. Therefore, the kinetics of the number of DEGs do not appear to be due to the adaptation of the skin to UV irradiation. There were more downregulated genes (514) than upregulated genes (123), which is consistent with the findings of a previous study [24].

In our DAVID GO analysis (Table1), the downregulated genes showed more functional diversity than the upregulated genes. Generally, photoaged skin tissue exhibits increased apoptosis and decreased ECM content [16, 22, 23]. Our GO analysis gene lists also include genes related to ‘negative regulation of apoptosis’ and ‘regulation of ECM’. Moreover, expressions of genes related to ‘positive regulation of cell communication’ and ‘positive regulation of signal transduction’ were decreased, implicating reduced cell–cell communication in the photoaging process (Table 1). The loss of cell communication may lead to a decrease in physiological functions in response to environmental stimuli [25]. Altered cell–cell communication is considered a hallmark of aging (intrinsic aging) [26]. These findings align with the understanding that the mechanisms of photoaging and intrinsic aging overlap with each other [10, 16]. Furthermore, we found downregulation of ’glycosaminoglycan binding’ and ’morphogenesis of epithelium’. Glycosaminoglycan is known to be associated with the maintenance of skin structure, and its supplementation can play a protective role in skin aging [27, 28]. Additionally, the crosstalk between keratinocytes and fibroblasts is known to be essential in maintaining epidermal stability and facilitating the wound repair process [29, 30].

Meanwhile, the GO analysis demonstrated that ‘immune response’ and ‘lipid biosynthesis process’ were upregulated. The immune response is well known to be associated with photoaging process. UV radiation itself can induce changes in immune cells [31]. Additionally, the pro-inflammatory effect of UV irradiation on the skin is well-recognized [32, 33]. Specifically, among the genes we identified (Table 1), *Cxcl10* is a monocyte/macrophage activation marker, and *Isg*15 is associated with LPS-induced inflammatory response [34, 35]. Inflammation development can be regarded as a process of aging from the perspective of “inflammaging” [36]. These evidence, along with our findings, supports the association of the immune response with the photoaging process. Lipid synthesis was thought to be decreased by UV irradiation in *in vitro* experiment [37]. However, a recent study showed UV-induced skin lipid and metabolite levels can vary depending on the gut microbiome status [38]. Further studies are necessary to understand the mechanisms of UV-associated lipid biosynthesis.

Through K-means clustering analysis, we found that genes could be categorized according to their expression patterns after UV irradiation. The genes involved in similar biological processes, molecular functions, and cellular components were enriched in the same clusters. Therefore, we hypothesized that genes that lead to photoaging phenotypes might show similar expression kinetics. The best-known genes associated with photoaging are collagen-regulation genes. So, we selected 11 seed genes associated with collagen synthesis, processing, or stability and identified the genes that have similar expression kinetics to those 11 genes through co-expression network analysis. The expected functions of the output genes were: 1) genes that regulate collagen function, 2) other ECM components associated with dermal aging, and 3) genes that are related to another photoaging phenotype coincident with wrinkling, such as dryness and tumor formation. Of the 504 genes we found in network analysis, the median ranking of the 11 seeding genes was 39 (S2 Table), which means that the results of the analysis were reliable and predictable. *Lum* was intentionally not used as a seed gene, although it is already known to be related to collagen fibril organization [39]. Surprisingly, *Lum* was ranked 41^st^ in our top 50 candidate genes list. This indirectly confirms the usefulness of the proposed list. Although there may be false-discovered genes, a considerable number of them may participate in the photoaging process. GO analysis of 50 highly ranked genes from a total of 504 genes showed that genes assigned to “morphogenesis of epithelium” were 4.74-fold enriched compared to those from the original 514 downregulated gene list. This suggests that in photoaging skin, genes involved in epidermal morphogenesis may also be involved in collagen regulation. *Lmo4*, *Tgif1*, *Chuk*, *Fgfr3*, *Igf1r*, and *Sfrp1* have been reported to play a role in epithelial cell proliferation and/or differentiation [40–47].

It is well recognized that proliferation and differentiation of epidermal keratinocytes are impaired in the elderly [48]. Given that, downregulation of these genes may lead to the dysregulation of proliferation and differentiation, resulting in skin barrier defects, which eventually lead to rough texture or dryness in photoaged skin.

In addition, we identified several genes (*Ccdc80*, *Fn1*, *Lum*, *Prelp*,*and Tgfbr3*) involved in the regulation of the extracellular matrix among the top 50 ranked genes. We successfully validated that the expressions of *Ccdc80*, *Fn1*, *Prelp*, and *Tgfbr3* are directly suppressed by UV irradiation in human fibroblasts, leading to downregulation of collagen synthesis (Fig 5). While previous studies have explored the functional role of these genes in collagen modulation, most of them focused on cardiac fibroblasts, chondrocytes, or endothelial cells, with no direct evidence of their collagen-regulatory function in human dermal fibroblasts associated with UV stimulation [18, 49–57]. Therefore, our results demonstrate the collagen-regulatory function of these genes (*Ccdc80*, *Fn1*, *Prelp*, and *Tgfbr3*) in the photoaging process. Additionally, these results validate the significance of the genes identified in understanding the mechanisms of photoaging.

In conclusion, our study utilized microarray analysis and advanced bioinformatics tools to identify several genes that were either up- or downregulated, directly or indirectly involved in ECM remodeling and inflammation. We also observed significant time-dependent kinetics in their expression levels following UV exposure. Furthermore, through network analysis, we identified gene lists that may play a potential role in the photoaging process. Among the numerous genes we identified, we directly validated that some of them were downregulated as a result of UV irradiation, subsequently leading to a reduction in collagen synthesis. While further investigation is required to uncover the specific functional roles of the remaining genes in the photoaging process, our data provides valuable insights into the complex mechanisms of photoaging. Moreover, our findings would provide meaningful hallmarks to explore time-dependent photoaging process using advance technology such as single cell RNA sequencing, or spatial transcriptomics analysis in future studies.

## Supporting information

S1 Fig Validation of the reliability of microarray analysis. Six genes were randomly selected, 3 from up-regulated, and 3 from down-regulated genes of the DEG lists (compared to 1 week control). Quantitative real-time PCR was performed. *Gasdermin C*, *Isg15*, *Saa3*, *Klf4*, *S100a3* and *Tesc* gene expression was significantly up or down-regulated as was the results from the microarray analysis.
(TIF)

S1 Table Primer sequences for quantitative RT-PCR. (DOCX)

S2 Table Predicted ranking of each collagen regulation genes through co-expression network constructed by 10 other known genes involving collagen regulation. (DOCX)
