# Supplementary figures and images for: Functionally similar genes exhibit comparable/similar time-course expression kinetics in the UV-induced photoaged mouse model
Source: PLoS One. 2023 Nov 9;18(11):e0290358. doi: 10.1371/journal.pone.0290358 (PMC10635544; doi:10.1371/journal.pone.0290358)

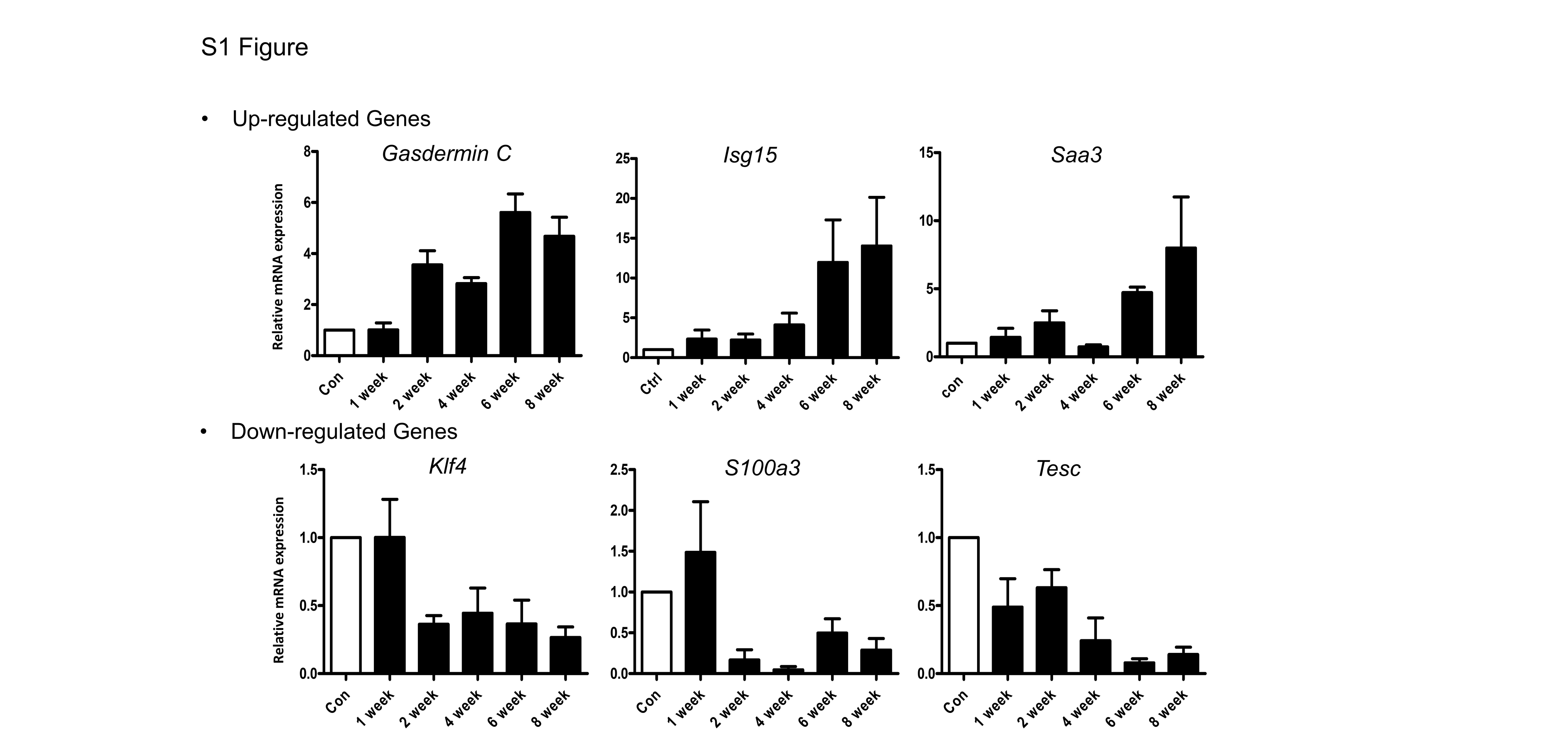

Supplement: S1 Fig — Six genes were randomly selected, 3 from up-regulated, and 3 from down-regulated genes of the DEG lists (compared to 1 week control). Quantitative real-time PCR was performed. Gasdermin C, Isg15, Saa3, Klf4, S100a3 and Tesc gene expression was significantly up or down-regulated as was the results from the microarray analysis. (TIF) [file pone.0290358.s001.tif]
