# Supplementary material for: Functionally similar genes exhibit comparable/similar time-course expression kinetics in the UV-induced photoaged mouse model
Source: PLoS One. 2023 Nov 9;18(11):e0290358. doi: 10.1371/journal.pone.0290358 (PMC10635544; doi:10.1371/journal.pone.0290358)
Supplement: S1 Table — (DOCX) [file pone.0290358.s002.docx]

**S1 Table**

|  | **Forward** | **Reverse** |
| --- | --- | --- |
| *Gasdermin C* | CTCCAGTTTCCTGCACATGCTC | GCTCATCCAGGATTTTGCCACC |
| *Isg15* | CATCCTGGTGAGGAACGAAAGG | CTCAGCCAGAACTGGTCTTCGT |
| *Saa3* | GAGCCTGGGCTGCTAAAGTCAT | CACTCATTGGCAAACTGGTCAGC |
| *Klf4* | GCGGGAAGGGAGAAGACA | CCGGATCGGATAGGTGAA |
| *Tesc* | ACAATGTCCCTGACCTGGAG | CCTTCTCCTTTCGTGACAGC |
| *S100a3* | AGATCTGCCAGTCGGAGCTCAA | GTCCACTTCGCAGTCTTTGTTGG |
| *36B4* | TCGACAATGGCAGCATCTAC | TGATGCAACAGTTGGGTAGC |
| *COL1A1* | CTCGAGGTGGACACCACCCT | CAGCTGGATGGCCACATCGG |
| *FN1* | GTGAAGAACGAGGAGGATGTG | GTGATGGCGGATGATGTAGC |
| *PRELP* | CATGGCCTCAAGAACCTCAT | AGCACAAGCAGGTTGGAGAT |
| *CCDC80* | AGCCCGGAGTACTTCTCCAT | CCATCCTGGTATCCTTGGTG |
| *LUM* | TGATCTGCAGTGGCTCATTC | AAAAGAGCCAGCTTTGTGA |
| *TGFBR3* | TAAACATGACCCTGGGCTTC | TGGATGTTCTCACCTGGACA |
