# Supplementary material for: Functionally similar genes exhibit comparable/similar time-course expression kinetics in the UV-induced photoaged mouse model
Source: PLoS One. 2023 Nov 9;18(11):e0290358. doi: 10.1371/journal.pone.0290358 (PMC10635544; doi:10.1371/journal.pone.0290358)
Supplement: S2 Table — (DOCX) [file pone.0290358.s003.docx]

**S2 Table**

| Gene | Predicted ranking | Optimal network threshold |
| --- | --- | --- |
| *SMOC2* | 1 | 0.946 |
| *COL4A2* | 2 | 0.946 |
| *SERPING1* | 17 | 0.948 |
| *PCOLCE2* | 30 | 0.947 |
| *SPARC* | 38 | 0.946 |
| *P4HA1* | 39 | 0.946 |
| *TGFBI* | 43 | 0.947 |
| *SERPINH1* | 51 | 0.948 |
| *LEPREL2* | 69 | 0.946 |
| *THBS1* | 125 | 0.939 |
| *COL11A1* | 261 | 0.946 |
